# (Dimethyl­formamide)(2-methyl­propen­oato)[tris­(1-methyl-1*H*-benzimidazol-2-ylmeth­yl)amine]­manganese(II) perchlorate dimethyl­formamide solvate

**DOI:** 10.1107/S1600536810036457

**Published:** 2010-09-18

**Authors:** Huilu Wu, Fei Jia, Jin Kong, Fan Kou, Jingkun Yuan

**Affiliations:** a School of Chemical and Biological Engineering, Lanzhou Jiaotong University, Lanzhou 730070, People’s Republic of China

## Abstract

In the title complex, [Mn(C_4_H_5_O_2_)(C_27_H_27_N_7_)(C_3_H_7_NO)]ClO_4_·C_3_H_7_NO, the Mn^II^ ion is seven-coordinated in a distorted monocapped trigonal-prismatic geometry formed by a tetra­dentate tris­(1-methyl-1*H*-benzimidazol-2-ylmeth­yl)amine mol­ecule, a bidentate 2-methacrylate anion and a dimethyl­formamide mol­ecule. The methyl groups of the coordinated dimethyl­formamide mol­ecule and the perchlorate anions are disordered over two positions with occupancy factors of 0.640 (8) and 0.360 (8).

## Related literature

For the biological activity of benzimidazole derivatives, see: Horton *et al.* (2003 ▶). For related structures, see: Wu *et al.* (2005 ▶, 2009 ▶).
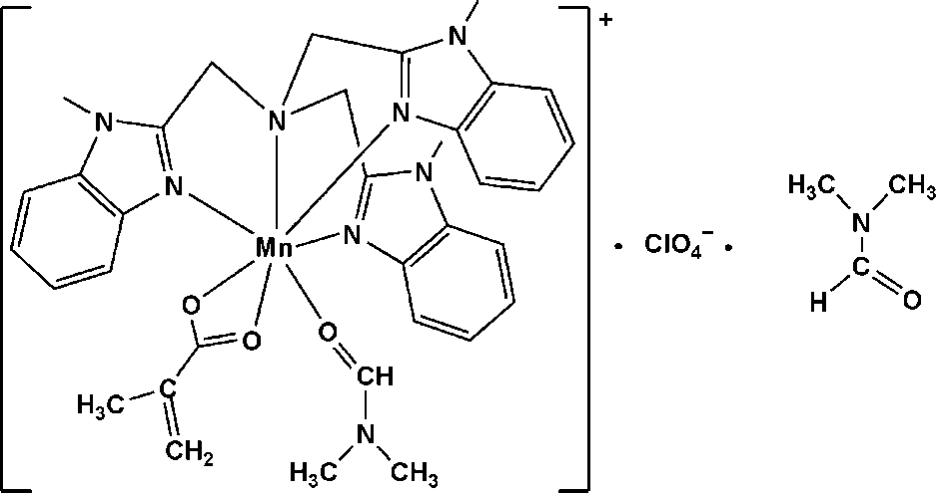


## Experimental

### 

#### Crystal data

[Mn(C_4_H_5_O_2_)(C_27_H_27_N_7_)(C_3_H_7_NO)]ClO_4_·C_3_H_7_NO
*M* *_r_* = 835.22
Triclinic, 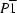

*a* = 12.139 (5) Å
*b* = 12.354 (5) Å
*c* = 15.146 (6) Å
α = 89.168 (4)°
β = 71.098 (4)°
γ = 76.936 (5)°
*V* = 2088.9 (14) Å^3^
*Z* = 2
Mo *K*α radiation
μ = 0.44 mm^−1^
*T* = 296 K
0.38 × 0.34 × 0.30 mm

#### Data collection

Bruker APEXII area-detector diffractometer
Absorption correction: multi-scan (*SADABS*: Bruker, 2006 ▶) *T*_min_ = 0.851, *T*_max_ = 0.880
15471 measured reflections
7680 independent reflections
5422 reflections with *I* > 2σ(*I*)
*R*_int_ = 0.033

#### Refinement

*R*[*F* ^2^ > 2σ(*F* ^2^)] = 0.059
*wR*(*F* ^2^) = 0.187
*S* = 1.10
7680 reflections
525 parameters
8 restraints
H-atom parameters constrained
Δρ_max_ = 0.90 e Å^−3^
Δρ_min_ = −0.54 e Å^−3^

### 

Data collection: *APEX2* (Bruker, 2006 ▶); cell refinement: *SAINT* (Bruker, 2006 ▶); data reduction: *SAINT*; program(s) used to solve structure: *SHELXS97* (Sheldrick, 2008 ▶); program(s) used to refine structure: *SHELXL97* (Sheldrick, 2008 ▶); molecular graphics: *SHELXTL* (Sheldrick, 2008 ▶); software used to prepare material for publication: *SHELXTL*.

## Supplementary Material

Crystal structure: contains datablocks global, I. DOI: 10.1107/S1600536810036457/is2590sup1.cif

Structure factors: contains datablocks I. DOI: 10.1107/S1600536810036457/is2590Isup2.hkl

Additional supplementary materials:  crystallographic information; 3D view; checkCIF report

## Figures and Tables

**Fig. 1.**
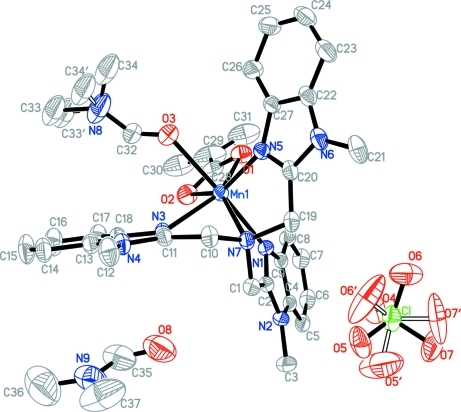
Molecular structure and atom numbering of the title compound with displacement ellipsoides drawn at the 30% probability level. H atoms bonded to C atoms have been omitted for clarity.

## supplementary crystallographic information

### Comment

The benzimidazole core is of a wide interest because of its diverse biological activities, and it is a well known structure in medicinal chemistry (Horton *et al.*, 2003). We are interested in tris(2-benzimidazolylmethyl)amine and its derivatives and previously reported the crystal structure of some related complexes (Wu *et al.*, 2009; Wu *et al.*, 2005).

The asymmetric unit of the title compound consists of a discrete [Mn(Mentb)(2-methacrylato)(DMF)] cations, a perchlorate anions, and one molecule of DMF (Fig. 1). The Mentb ligand forms a tripodal pyramidal geometry with the manganese ion, and the remaining open axial site of the complex is occupied by a chelating 2-methacrylate anion and a monodentate DMF. The complex has a seven-coordinate manganese(II) geometry with a N_4_O_3_ donor set.

### Experimental

To a stirred solution of Mentb (0.5 mmol) in hot methanol (20 ml) was added Mn(ClO_4_)_2_.6 H_2_O (0.5 mmol) followed by a solution of Na(2-methacrylate) (0.5 mmol) in methanol (5 ml), whereupon a colorless microcrystalline precipitate was produced and collected by filtration. After drying in air the colorless product was redissolved in DMF/methanol (1:1) and filtered. Light-yellow block-shaped crystals suitable for X-ray diffraction studies were obtained by vapor diffusion of diethyl ether into the filtrate for 4 weeks at room temperature (yield 0.34 g, 82%). Elemental analysis found: C 53.21, H 5.55, N 15.09%; calcd. for C_37_H_46_N_9_O_8_MnCl: C 53.04, H 5.39, N 15.13%.

### Refinement

H atoms bonded to C atoms were placed in calculated positions and included in a riding-model approximation, with C—H distances ranging from 0.93 to 0.97 Å and *U*_iso_(H) = 1.2*U*_eq_(C) or *U*_iso_(H) = 1.5*U*_eq_(C_methyl_). The coordinated DMF molecule and the perchlorate anion are disordered over two positions with occupancy factors in the ratio 16/9. In the refinement, several constrains were applied: *EADP* for atoms C29 C30 C31 and for atoms N8 C33 C34 C33' C34', *SADI* for atoms N8 C33 C34 C33' C34', *DELU* for atoms MN1 O2.

### Crystal data

**Table:**

[Mn(C_4_H_5_O_2_)(C_27_H_27_N_7_)(C_3_H_7_NO)]ClO_4_·C_3_H_7_NO	*Z* = 2
*M**_r_* = 835.22	*F*(000) = 874
Triclinic, *P*1	*D*_x_ = 1.328 Mg m^−^^3^
Hall symbol: -P 1	Mo *K*α radiation, λ = 0.71073 Å
*a* = 12.139 (5) Å	Cell parameters from 6078 reflections
*b* = 12.354 (5) Å	θ = 2.3–26.9°
*c* = 15.146 (6) Å	µ = 0.44 mm^−^^1^
α = 89.168 (4)°	*T* = 296 K
β = 71.098 (4)°	Block, light-yellow
γ = 76.936 (5)°	0.38 × 0.34 × 0.30 mm
*V* = 2088.9 (14) Å^3^	

### Data collection

**Table:**

Bruker APEXII area-detector diffractometer	7680 independent reflections
Radiation source: fine-focus sealed tube	5422 reflections with *I* > 2σ(*I*)
graphite	*R*_int_ = 0.033
ω scans	θ_max_ = 25.5°, θ_min_ = 1.7°
Absorption correction: multi-scan (*SADABS*: Bruker, 2006)	*h* = −14→14
*T*_min_ = 0.851, *T*_max_ = 0.880	*k* = −14→13
15471 measured reflections	*l* = −18→18

### Refinement

**Table:**

Refinement on *F*^2^	Primary atom site location: structure-invariant direct methods
Least-squares matrix: full	Secondary atom site location: difference Fourier map
*R*[*F*^2^ > 2σ(*F*^2^)] = 0.059	Hydrogen site location: inferred from neighbouring sites
*wR*(*F*^2^) = 0.187	H-atom parameters constrained
*S* = 1.10	*w* = 1/[σ^2^(*F*_o_^2^) + (0.1111*P*)^2^ + 0.1787*P*] where *P* = (*F*_o_^2^ + 2*F*_c_^2^)/3
7680 reflections	(Δ/σ)_max_ = 0.001
525 parameters	Δρ_max_ = 0.90 e Å^−^^3^
8 restraints	Δρ_min_ = −0.54 e Å^−^^3^

### Special details

**Table:**

Geometry. All e.s.d.'s (except the e.s.d. in the dihedral angle between two l.s. planes) are estimated using the full covariance matrix. The cell e.s.d.'s are taken into account individually in the estimation of e.s.d.'s in distances, angles and torsion angles; correlations between e.s.d.'s in cell parameters are only used when they are defined by crystal symmetry. An approximate (isotropic) treatment of cell e.s.d.'s is used for estimating e.s.d.'s involving l.s. planes.
Refinement. Refinement of *F*^2^ against ALL reflections. The weighted *R*-factor *wR* and goodness of fit *S* are based on *F*^2^, conventional *R*-factors *R* are based on *F*, with *F* set to zero for negative *F*^2^. The threshold expression of *F*^2^ > σ(*F*^2^) is used only for calculating *R*-factors(gt) *etc*. and is not relevant to the choice of reflections for refinement. *R*-factors based on *F*^2^ are statistically about twice as large as those based on *F*, and *R*- factors based on ALL data will be even larger.

### Fractional atomic coordinates and isotropic or equivalent isotropic displacement parameters (Å^2^)

**Table:**

	*x*	*y*	*z*	*U*_iso_*/*U*_eq_	Occ. (<1)
Mn1	0.78941 (4)	0.30677 (4)	0.72233 (3)	0.04338 (18)	
Cl	0.42500 (11)	0.73755 (8)	0.62722 (8)	0.0706 (3)	
O1	0.9336 (2)	0.3582 (2)	0.76647 (19)	0.0655 (7)	
O2	0.8252 (3)	0.2580 (2)	0.86003 (18)	0.0720 (8)	
O3	0.9336 (3)	0.1338 (2)	0.6630 (2)	0.0757 (8)	
O4	0.4724 (4)	0.7215 (3)	0.7008 (3)	0.1208 (14)	
O5	0.3586 (10)	0.6632 (8)	0.6261 (7)	0.101 (3)	0.640 (8)
O5'	0.3062 (18)	0.740 (4)	0.669 (2)	0.262 (15)	0.360 (8)
O6	0.5290 (5)	0.7232 (11)	0.5421 (4)	0.111 (3)	0.640 (8)
O6'	0.463 (3)	0.6437 (14)	0.5688 (19)	0.190 (13)	0.360 (8)
O7	0.3568 (13)	0.8430 (9)	0.6244 (10)	0.131 (5)	0.640 (8)
O7'	0.423 (3)	0.8347 (19)	0.5935 (19)	0.172 (16)	0.360 (8)
O8	0.3489 (4)	0.2371 (5)	0.7818 (4)	0.1465 (19)	
N1	0.6526 (2)	0.4681 (2)	0.79600 (18)	0.0437 (6)	
N2	0.4589 (2)	0.5539 (2)	0.85885 (19)	0.0494 (7)	
N3	0.6674 (3)	0.1841 (2)	0.74261 (18)	0.0477 (6)	
N4	0.6038 (3)	0.0753 (2)	0.66356 (19)	0.0524 (7)	
N5	0.8692 (2)	0.3485 (2)	0.57493 (18)	0.0473 (6)	
N6	0.8642 (3)	0.4395 (2)	0.4480 (2)	0.0581 (8)	
N7	0.6286 (2)	0.3665 (2)	0.64674 (18)	0.0451 (6)	
N8	1.0068 (5)	−0.0478 (4)	0.6781 (4)	0.1245 (16)	
N9	0.2678 (5)	0.0927 (5)	0.7980 (4)	0.131 (2)	
C1	0.5169 (3)	0.4191 (3)	0.7206 (2)	0.0513 (8)	
H1A	0.4758	0.3625	0.7501	0.062*	
H1B	0.4649	0.4698	0.6935	0.062*	
C2	0.5432 (3)	0.4806 (3)	0.7911 (2)	0.0448 (7)	
C3	0.3310 (3)	0.5872 (4)	0.8722 (3)	0.0681 (10)	
H3A	0.3158	0.6501	0.8362	0.102*	
H3B	0.2871	0.6071	0.9372	0.102*	
H3C	0.3059	0.5265	0.8519	0.102*	
C4	0.5162 (3)	0.5930 (3)	0.9121 (2)	0.0478 (8)	
C5	0.4734 (4)	0.6652 (3)	0.9913 (2)	0.0601 (10)	
H5	0.3921	0.6975	1.0184	0.072*	
C6	0.5565 (4)	0.6868 (3)	1.0279 (2)	0.0631 (10)	
H6	0.5309	0.7347	1.0812	0.076*	
C7	0.6773 (4)	0.6392 (3)	0.9874 (3)	0.0639 (10)	
H7	0.7313	0.6573	1.0132	0.077*	
C8	0.7203 (3)	0.5645 (3)	0.9086 (2)	0.0538 (8)	
H8	0.8017	0.5323	0.8819	0.065*	
C9	0.6373 (3)	0.5401 (3)	0.8718 (2)	0.0438 (7)	
C10	0.6214 (3)	0.2630 (3)	0.6052 (2)	0.0517 (8)	
H10A	0.6855	0.2427	0.5459	0.062*	
H10B	0.5456	0.2733	0.5939	0.062*	
C11	0.6318 (3)	0.1731 (3)	0.6710 (2)	0.0454 (7)	
C12	0.5611 (4)	0.0362 (3)	0.5929 (3)	0.0752 (12)	
H12A	0.5215	0.0991	0.5677	0.113*	
H12B	0.5060	−0.0092	0.6209	0.113*	
H12C	0.6279	−0.0068	0.5436	0.113*	
C13	0.6210 (3)	0.0157 (3)	0.7386 (2)	0.0541 (8)	
C14	0.6035 (4)	−0.0882 (3)	0.7658 (3)	0.0721 (11)	
H14	0.5785	−0.1335	0.7314	0.086*	
C15	0.6253 (5)	−0.1200 (4)	0.8462 (3)	0.0934 (16)	
H15	0.6138	−0.1888	0.8678	0.112*	
C16	0.6642 (5)	−0.0528 (4)	0.8971 (3)	0.0887 (15)	
H16	0.6786	−0.0784	0.9513	0.106*	
C17	0.6822 (4)	0.0506 (3)	0.8695 (3)	0.0672 (11)	
H17	0.7077	0.0954	0.9040	0.081*	
C18	0.6601 (3)	0.0845 (3)	0.7875 (2)	0.0523 (8)	
C19	0.6679 (3)	0.4447 (3)	0.5758 (2)	0.0509 (8)	
H19A	0.6445	0.5197	0.6045	0.061*	
H19B	0.6306	0.4440	0.5281	0.061*	
C20	0.8004 (3)	0.4101 (3)	0.5331 (2)	0.0479 (8)	
C21	0.8145 (5)	0.5073 (4)	0.3826 (3)	0.0786 (13)	
H21A	0.8689	0.5514	0.3497	0.118*	
H21B	0.7388	0.5554	0.4169	0.118*	
H21C	0.8036	0.4591	0.3385	0.118*	
C22	0.9825 (4)	0.3943 (3)	0.4344 (2)	0.0570 (9)	
C23	1.0863 (5)	0.3980 (4)	0.3602 (3)	0.0747 (12)	
H23	1.0833	0.4373	0.3079	0.090*	
C24	1.1928 (5)	0.3405 (4)	0.3687 (3)	0.0857 (14)	
H24	1.2636	0.3402	0.3205	0.103*	
C25	1.1982 (4)	0.2829 (4)	0.4467 (3)	0.0795 (12)	
H25	1.2724	0.2454	0.4499	0.095*	
C26	1.0948 (4)	0.2799 (3)	0.5206 (3)	0.0679 (10)	
H26	1.0985	0.2408	0.5730	0.081*	
C27	0.9862 (3)	0.3374 (3)	0.5132 (2)	0.0517 (8)	
C28	0.9084 (3)	0.3102 (3)	0.8409 (3)	0.0598 (10)	
C29	0.9747 (6)	0.3139 (7)	0.9058 (4)	0.1252 (15)	
C31	1.0517 (5)	0.3945 (6)	0.8873 (4)	0.1252 (15)	
H31A	1.0836	0.3965	0.9374	0.188*	
H31B	1.0051	0.4671	0.8830	0.188*	
H31C	1.1162	0.3721	0.8295	0.188*	
C30	0.9638 (5)	0.2499 (6)	0.9765 (4)	0.1252 (15)	
H30A	0.9134	0.2011	0.9860	0.150*	
H30B	1.0065	0.2532	1.0171	0.150*	
C32	0.9487 (4)	0.0529 (4)	0.7078 (4)	0.0821 (13)	
H32	0.9137	0.0652	0.7723	0.099*	
C33	1.0101 (11)	−0.1517 (6)	0.7264 (8)	0.1245 (16)	0.640 (8)
H33A	0.9790	−0.1347	0.7928	0.187*	0.640 (8)
H33B	1.0913	−0.1945	0.7094	0.187*	0.640 (8)
H33C	0.9623	−0.1938	0.7087	0.187*	0.640 (8)
C33'	1.0174 (19)	−0.1210 (11)	0.7540 (9)	0.1245 (16)	0.360 (8)
H33D	1.0376	−0.0826	0.7992	0.187*	0.360 (8)
H33E	1.0790	−0.1872	0.7287	0.187*	0.360 (8)
H33F	0.9425	−0.1409	0.7838	0.187*	0.360 (8)
C34	1.0828 (9)	−0.0635 (7)	0.5788 (6)	0.1245 (16)	0.640 (8)
H34A	1.0330	−0.0483	0.5400	0.187*	0.640 (8)
H34B	1.1294	−0.1389	0.5655	0.187*	0.640 (8)
H34C	1.1353	−0.0135	0.5664	0.187*	0.640 (8)
C34'	1.0205 (19)	−0.1070 (12)	0.5909 (9)	0.1245 (16)	0.360 (8)
H34D	0.9432	−0.1006	0.5845	0.187*	0.360 (8)
H34E	1.0577	−0.1841	0.5919	0.187*	0.360 (8)
H34F	1.0697	−0.0751	0.5390	0.187*	0.360 (8)
C35	0.3276 (6)	0.1548 (7)	0.8206 (5)	0.122 (2)	
H35	0.3562	0.1350	0.8701	0.146*	
C36	0.2514 (11)	−0.0045 (10)	0.8492 (8)	0.230 (6)	
H36A	0.3055	−0.0197	0.8846	0.345*	
H36B	0.2674	−0.0670	0.8063	0.345*	
H36C	0.1704	0.0083	0.8910	0.345*	
C37	0.2181 (7)	0.1126 (8)	0.7233 (7)	0.190 (5)	
H37A	0.1323	0.1307	0.7489	0.286*	
H37B	0.2447	0.0470	0.6818	0.286*	
H37C	0.2442	0.1735	0.6894	0.286*	

### Atomic displacement parameters (Å^2^)

**Table:**

	*U*^11^	*U*^22^	*U*^33^	*U*^12^	*U*^13^	*U*^23^
Mn1	0.0455 (3)	0.0413 (3)	0.0407 (3)	−0.0102 (2)	−0.0107 (2)	0.0032 (2)
Cl	0.0939 (8)	0.0588 (6)	0.0796 (7)	−0.0275 (6)	−0.0500 (7)	0.0179 (5)
O1	0.0589 (16)	0.0718 (18)	0.0626 (16)	−0.0108 (14)	−0.0186 (14)	−0.0053 (14)
O2	0.0749 (19)	0.0780 (19)	0.0576 (14)	−0.0080 (16)	−0.0216 (14)	0.0110 (13)
O3	0.0718 (19)	0.0590 (18)	0.085 (2)	−0.0056 (14)	−0.0164 (16)	0.0000 (15)
O4	0.178 (4)	0.139 (3)	0.104 (3)	−0.088 (3)	−0.092 (3)	0.047 (2)
O5	0.133 (7)	0.091 (6)	0.129 (6)	−0.078 (5)	−0.076 (6)	0.039 (5)
O5'	0.100 (13)	0.31 (4)	0.31 (3)	0.021 (18)	−0.026 (16)	0.07 (3)
O6	0.066 (4)	0.176 (9)	0.081 (4)	−0.014 (4)	−0.020 (3)	0.017 (5)
O6'	0.31 (3)	0.089 (11)	0.21 (2)	0.070 (15)	−0.21 (2)	−0.072 (13)
O7	0.165 (9)	0.061 (6)	0.132 (8)	0.018 (7)	−0.033 (7)	0.007 (5)
O7'	0.36 (4)	0.11 (2)	0.15 (2)	−0.15 (3)	−0.17 (3)	0.093 (17)
O8	0.106 (3)	0.148 (5)	0.172 (5)	−0.058 (3)	−0.007 (3)	−0.030 (4)
N1	0.0414 (15)	0.0390 (14)	0.0439 (14)	−0.0062 (12)	−0.0071 (12)	0.0013 (11)
N2	0.0401 (15)	0.0471 (16)	0.0472 (15)	−0.0060 (13)	0.0012 (13)	0.0081 (13)
N3	0.0549 (17)	0.0387 (15)	0.0468 (15)	−0.0130 (13)	−0.0122 (14)	0.0066 (12)
N4	0.0658 (19)	0.0438 (16)	0.0499 (16)	−0.0199 (14)	−0.0172 (15)	0.0031 (13)
N5	0.0503 (16)	0.0414 (15)	0.0434 (14)	−0.0120 (13)	−0.0053 (13)	0.0017 (12)
N6	0.077 (2)	0.0525 (18)	0.0455 (16)	−0.0262 (16)	−0.0139 (16)	0.0113 (13)
N7	0.0509 (16)	0.0371 (14)	0.0464 (15)	−0.0133 (12)	−0.0131 (13)	0.0079 (11)
N8	0.130 (3)	0.060 (2)	0.192 (4)	0.000 (2)	−0.078 (3)	−0.022 (3)
N9	0.098 (4)	0.134 (5)	0.145 (5)	−0.048 (3)	−0.003 (3)	−0.058 (4)
C1	0.0450 (19)	0.047 (2)	0.061 (2)	−0.0115 (15)	−0.0166 (17)	0.0082 (16)
C2	0.0453 (19)	0.0389 (17)	0.0443 (17)	−0.0098 (14)	−0.0073 (15)	0.0099 (14)
C3	0.044 (2)	0.071 (3)	0.072 (3)	−0.0028 (19)	−0.0036 (19)	0.010 (2)
C4	0.055 (2)	0.0366 (17)	0.0406 (17)	−0.0086 (15)	−0.0026 (16)	0.0096 (14)
C5	0.070 (3)	0.044 (2)	0.0439 (19)	−0.0052 (18)	0.0059 (19)	0.0069 (15)
C6	0.091 (3)	0.045 (2)	0.0378 (18)	−0.007 (2)	−0.007 (2)	−0.0021 (15)
C7	0.084 (3)	0.056 (2)	0.052 (2)	−0.014 (2)	−0.024 (2)	0.0008 (17)
C8	0.056 (2)	0.050 (2)	0.0482 (19)	−0.0062 (16)	−0.0115 (17)	0.0012 (16)
C9	0.0522 (19)	0.0360 (17)	0.0358 (16)	−0.0097 (14)	−0.0048 (15)	0.0055 (13)
C10	0.067 (2)	0.0474 (19)	0.0470 (18)	−0.0198 (17)	−0.0222 (18)	0.0062 (15)
C11	0.0483 (19)	0.0400 (17)	0.0454 (17)	−0.0137 (15)	−0.0096 (15)	0.0022 (14)
C12	0.107 (4)	0.060 (2)	0.071 (3)	−0.037 (2)	−0.033 (3)	0.000 (2)
C13	0.062 (2)	0.0429 (19)	0.0512 (19)	−0.0149 (17)	−0.0080 (18)	0.0042 (15)
C14	0.093 (3)	0.047 (2)	0.073 (3)	−0.028 (2)	−0.016 (2)	0.0063 (19)
C15	0.147 (5)	0.055 (3)	0.078 (3)	−0.039 (3)	−0.027 (3)	0.026 (2)
C16	0.139 (5)	0.066 (3)	0.060 (3)	−0.030 (3)	−0.027 (3)	0.026 (2)
C17	0.091 (3)	0.055 (2)	0.055 (2)	−0.018 (2)	−0.022 (2)	0.0100 (18)
C18	0.055 (2)	0.0433 (19)	0.0521 (19)	−0.0105 (16)	−0.0105 (17)	0.0093 (15)
C19	0.066 (2)	0.0404 (18)	0.0482 (18)	−0.0148 (16)	−0.0204 (18)	0.0109 (14)
C20	0.063 (2)	0.0402 (18)	0.0401 (17)	−0.0207 (16)	−0.0112 (17)	0.0035 (14)
C21	0.108 (4)	0.079 (3)	0.059 (2)	−0.043 (3)	−0.028 (2)	0.032 (2)
C22	0.073 (3)	0.0433 (19)	0.0475 (19)	−0.0252 (18)	−0.0015 (19)	−0.0051 (15)
C23	0.093 (3)	0.065 (3)	0.055 (2)	−0.040 (3)	0.005 (2)	−0.0015 (19)
C24	0.082 (3)	0.072 (3)	0.081 (3)	−0.040 (3)	0.019 (3)	−0.010 (2)
C25	0.058 (3)	0.071 (3)	0.093 (3)	−0.019 (2)	0.001 (2)	−0.009 (3)
C26	0.061 (3)	0.059 (2)	0.073 (3)	−0.015 (2)	−0.007 (2)	−0.006 (2)
C27	0.056 (2)	0.0402 (18)	0.0497 (19)	−0.0172 (16)	−0.0013 (17)	−0.0059 (15)
C28	0.048 (2)	0.064 (2)	0.059 (2)	0.0026 (19)	−0.0163 (19)	−0.0164 (19)
C29	0.091 (2)	0.182 (4)	0.095 (2)	0.011 (2)	−0.048 (2)	−0.026 (2)
C31	0.091 (2)	0.182 (4)	0.095 (2)	0.011 (2)	−0.048 (2)	−0.026 (2)
C30	0.091 (2)	0.182 (4)	0.095 (2)	0.011 (2)	−0.048 (2)	−0.026 (2)
C32	0.067 (3)	0.054 (3)	0.113 (4)	−0.011 (2)	−0.015 (3)	0.001 (3)
C33	0.130 (3)	0.060 (2)	0.192 (4)	0.000 (2)	−0.078 (3)	−0.022 (3)
C33'	0.130 (3)	0.060 (2)	0.192 (4)	0.000 (2)	−0.078 (3)	−0.022 (3)
C34	0.130 (3)	0.060 (2)	0.192 (4)	0.000 (2)	−0.078 (3)	−0.022 (3)
C34'	0.130 (3)	0.060 (2)	0.192 (4)	0.000 (2)	−0.078 (3)	−0.022 (3)
C35	0.098 (5)	0.147 (7)	0.112 (5)	−0.042 (5)	−0.010 (4)	−0.047 (5)
C36	0.237 (13)	0.191 (11)	0.210 (11)	−0.105 (10)	0.035 (9)	−0.064 (9)
C37	0.131 (6)	0.195 (9)	0.251 (10)	0.021 (6)	−0.106 (7)	−0.111 (8)

### Geometric parameters (Å, °)

**Table:**

Mn1—N5	2.231 (3)	C10—C11	1.493 (4)
Mn1—O1	2.278 (3)	C10—H10A	0.9700
Mn1—N3	2.301 (3)	C10—H10B	0.9700
Mn1—O2	2.310 (3)	C12—H12A	0.9600
Mn1—N1	2.311 (3)	C12—H12B	0.9600
Mn1—O3	2.405 (3)	C12—H12C	0.9600
Mn1—N7	2.538 (3)	C13—C14	1.384 (5)
Cl—O7'	1.30 (2)	C13—C18	1.388 (5)
Cl—O5	1.356 (5)	C14—C15	1.362 (6)
Cl—O6'	1.365 (12)	C14—H14	0.9300
Cl—O5'	1.37 (2)	C15—C16	1.392 (7)
Cl—O7	1.385 (11)	C15—H15	0.9300
Cl—O4	1.403 (3)	C16—C17	1.380 (6)
Cl—O6	1.462 (6)	C16—H16	0.9300
O1—C28	1.248 (5)	C17—C18	1.393 (5)
O2—C28	1.272 (5)	C17—H17	0.9300
O3—C32	1.212 (5)	C19—C20	1.487 (5)
O8—C35	1.203 (9)	C19—H19A	0.9700
N1—C2	1.328 (4)	C19—H19B	0.9700
N1—C9	1.401 (4)	C21—H21A	0.9600
N2—C2	1.359 (4)	C21—H21B	0.9600
N2—C4	1.377 (4)	C21—H21C	0.9600
N2—C3	1.460 (4)	C22—C27	1.386 (5)
N3—C11	1.310 (4)	C22—C23	1.400 (6)
N3—C18	1.403 (4)	C23—C24	1.373 (7)
N4—C11	1.343 (4)	C23—H23	0.9300
N4—C13	1.391 (4)	C24—C25	1.382 (7)
N4—C12	1.461 (4)	C24—H24	0.9300
N5—C20	1.312 (4)	C25—C26	1.393 (6)
N5—C27	1.401 (4)	C25—H25	0.9300
N6—C20	1.364 (4)	C26—C27	1.388 (5)
N6—C22	1.365 (5)	C26—H26	0.9300
N6—C21	1.477 (5)	C28—C29	1.464 (6)
N7—C10	1.465 (4)	C29—C30	1.312 (10)
N7—C1	1.466 (4)	C29—C31	1.478 (10)
N7—C19	1.471 (4)	C31—H31A	0.9600
N8—C32	1.289 (6)	C31—H31B	0.9600
N8—C34'	1.462 (7)	C31—H31C	0.9600
N8—C33	1.467 (6)	C30—H30A	0.9300
N8—C33'	1.473 (7)	C30—H30B	0.9300
N8—C34	1.476 (7)	C32—H32	0.9300
N9—C35	1.286 (7)	C33—H33A	0.9600
N9—C36	1.437 (12)	C33—H33B	0.9600
N9—C37	1.439 (10)	C33—H33C	0.9600
C1—C2	1.475 (5)	C33'—H33D	0.9600
C1—H1A	0.9700	C33'—H33E	0.9600
C1—H1B	0.9700	C33'—H33F	0.9600
C3—H3A	0.9600	C34—H34A	0.9600
C3—H3B	0.9600	C34—H34B	0.9600
C3—H3C	0.9600	C34—H34C	0.9600
C4—C5	1.387 (5)	C34'—H34D	0.9600
C4—C9	1.399 (5)	C34'—H34E	0.9600
C5—C6	1.373 (6)	C34'—H34F	0.9600
C5—H5	0.9300	C35—H35	0.9300
C6—C7	1.382 (6)	C36—H36A	0.9600
C6—H6	0.9300	C36—H36B	0.9600
C7—C8	1.400 (5)	C36—H36C	0.9600
C7—H7	0.9300	C37—H37A	0.9600
C8—C9	1.387 (5)	C37—H37B	0.9600
C8—H8	0.9300	C37—H37C	0.9600
			
N5—Mn1—O1	92.23 (10)	N4—C11—C10	123.6 (3)
N5—Mn1—N3	115.22 (10)	N4—C12—H12A	109.5
O1—Mn1—N3	145.55 (10)	N4—C12—H12B	109.5
N5—Mn1—O2	145.57 (11)	H12A—C12—H12B	109.5
O1—Mn1—O2	56.72 (11)	N4—C12—H12C	109.5
N3—Mn1—O2	90.47 (10)	H12A—C12—H12C	109.5
N5—Mn1—N1	105.01 (10)	H12B—C12—H12C	109.5
O1—Mn1—N1	90.47 (10)	C14—C13—C18	123.6 (3)
N3—Mn1—N1	101.20 (10)	C14—C13—N4	131.2 (3)
O2—Mn1—N1	90.95 (10)	C18—C13—N4	105.3 (3)
N5—Mn1—O3	80.88 (11)	C15—C14—C13	115.8 (4)
O1—Mn1—O3	86.30 (10)	C15—C14—H14	122.1
N3—Mn1—O3	78.50 (10)	C13—C14—H14	122.1
O2—Mn1—O3	82.48 (11)	C14—C15—C16	122.1 (4)
N1—Mn1—O3	173.41 (9)	C14—C15—H15	119.0
N5—Mn1—N7	69.34 (10)	C16—C15—H15	119.0
O1—Mn1—N7	145.78 (10)	C17—C16—C15	122.0 (4)
N3—Mn1—N7	67.56 (9)	C17—C16—H16	119.0
O2—Mn1—N7	144.83 (10)	C15—C16—H16	119.0
N1—Mn1—N7	68.32 (9)	C16—C17—C18	116.7 (4)
O3—Mn1—N7	117.15 (10)	C16—C17—H17	121.7
O7'—Cl—O6'	120.4 (15)	C18—C17—H17	121.7
O7'—Cl—O5'	102.9 (18)	C13—C18—C17	119.9 (3)
O6'—Cl—O5'	101.4 (18)	C13—C18—N3	109.6 (3)
O5—Cl—O7	107.9 (8)	C17—C18—N3	130.5 (3)
O7'—Cl—O4	114.3 (9)	N7—C19—C20	108.4 (3)
O5—Cl—O4	111.4 (3)	N7—C19—H19A	110.0
O6'—Cl—O4	110.8 (5)	C20—C19—H19A	110.0
O5'—Cl—O4	104.3 (13)	N7—C19—H19B	110.0
O7—Cl—O4	116.3 (7)	C20—C19—H19B	110.0
O5—Cl—O6	111.2 (5)	H19A—C19—H19B	108.4
O7—Cl—O6	104.7 (6)	N5—C20—N6	112.4 (3)
O4—Cl—O6	105.2 (3)	N5—C20—C19	123.0 (3)
C28—O1—Mn1	92.8 (2)	N6—C20—C19	124.6 (3)
C28—O2—Mn1	90.7 (2)	N6—C21—H21A	109.5
C32—O3—Mn1	125.4 (3)	N6—C21—H21B	109.5
C2—N1—C9	104.4 (3)	H21A—C21—H21B	109.5
C2—N1—Mn1	115.8 (2)	N6—C21—H21C	109.5
C9—N1—Mn1	135.5 (2)	H21A—C21—H21C	109.5
C2—N2—C4	107.6 (3)	H21B—C21—H21C	109.5
C2—N2—C3	126.6 (3)	N6—C22—C27	106.0 (3)
C4—N2—C3	125.8 (3)	N6—C22—C23	131.8 (4)
C11—N3—C18	104.4 (3)	C27—C22—C23	122.2 (4)
C11—N3—Mn1	113.6 (2)	C24—C23—C22	116.5 (4)
C18—N3—Mn1	135.2 (2)	C24—C23—H23	121.7
C11—N4—C13	106.8 (3)	C22—C23—H23	121.7
C11—N4—C12	128.2 (3)	C23—C24—C25	122.1 (4)
C13—N4—C12	125.0 (3)	C23—C24—H24	118.9
C20—N5—C27	105.0 (3)	C25—C24—H24	118.9
C20—N5—Mn1	119.5 (2)	C24—C25—C26	121.3 (5)
C27—N5—Mn1	134.8 (2)	C24—C25—H25	119.4
C20—N6—C22	107.3 (3)	C26—C25—H25	119.4
C20—N6—C21	126.3 (3)	C27—C26—C25	117.5 (4)
C22—N6—C21	126.4 (3)	C27—C26—H26	121.3
C10—N7—C1	112.4 (3)	C25—C26—H26	121.3
C10—N7—C19	112.2 (2)	C22—C27—C26	120.4 (4)
C1—N7—C19	111.6 (3)	C22—C27—N5	109.2 (3)
C10—N7—Mn1	104.70 (18)	C26—C27—N5	130.3 (3)
C1—N7—Mn1	107.67 (18)	O1—C28—O2	119.7 (3)
C19—N7—Mn1	107.78 (19)	O1—C28—C29	119.9 (5)
C32—N8—C34'	128.0 (7)	O2—C28—C29	120.3 (5)
C32—N8—C33	130.8 (7)	C30—C29—C28	121.0 (7)
C32—N8—C33'	113.2 (8)	C30—C29—C31	122.9 (5)
C34'—N8—C33'	114.1 (7)	C28—C29—C31	116.1 (6)
C32—N8—C34	115.8 (6)	C29—C31—H31A	109.5
C33—N8—C34	113.4 (6)	C29—C31—H31B	109.5
C35—N9—C36	118.1 (9)	H31A—C31—H31B	109.5
C35—N9—C37	124.4 (8)	C29—C31—H31C	109.5
C36—N9—C37	117.6 (7)	H31A—C31—H31C	109.5
N7—C1—C2	110.0 (3)	H31B—C31—H31C	109.5
N7—C1—H1A	109.7	C29—C30—H30A	120.0
C2—C1—H1A	109.7	C29—C30—H30B	120.0
N7—C1—H1B	109.7	H30A—C30—H30B	120.0
C2—C1—H1B	109.7	O3—C32—N8	128.8 (6)
H1A—C1—H1B	108.2	O3—C32—H32	115.6
N1—C2—N2	112.8 (3)	N8—C32—H32	115.6
N1—C2—C1	123.1 (3)	N8—C33—H33A	109.5
N2—C2—C1	124.1 (3)	N8—C33—H33B	109.5
N2—C3—H3A	109.5	N8—C33—H33C	109.5
N2—C3—H3B	109.5	N8—C33'—H33D	109.5
H3A—C3—H3B	109.5	N8—C33'—H33E	109.5
N2—C3—H3C	109.5	H33D—C33'—H33E	109.5
H3A—C3—H3C	109.5	N8—C33'—H33F	109.5
H3B—C3—H3C	109.5	H33D—C33'—H33F	109.5
N2—C4—C5	131.9 (3)	H33E—C33'—H33F	109.5
N2—C4—C9	105.3 (3)	N8—C34—H34A	109.5
C5—C4—C9	122.8 (3)	N8—C34—H34B	109.5
C6—C5—C4	116.9 (4)	N8—C34—H34C	109.5
C6—C5—H5	121.6	N8—C34'—H34D	109.5
C4—C5—H5	121.6	N8—C34'—H34E	109.5
C5—C6—C7	121.6 (3)	H34D—C34'—H34E	109.5
C5—C6—H6	119.2	N8—C34'—H34F	109.5
C7—C6—H6	119.2	H34D—C34'—H34F	109.5
C6—C7—C8	121.6 (4)	H34E—C34'—H34F	109.5
C6—C7—H7	119.2	O8—C35—N9	124.0 (8)
C8—C7—H7	119.2	O8—C35—H35	118.0
C9—C8—C7	117.6 (4)	N9—C35—H35	118.0
C9—C8—H8	121.2	N9—C36—H36A	109.5
C7—C8—H8	121.2	N9—C36—H36B	109.5
C8—C9—C4	119.5 (3)	H36A—C36—H36B	109.5
C8—C9—N1	130.6 (3)	N9—C36—H36C	109.5
C4—C9—N1	109.9 (3)	H36A—C36—H36C	109.5
N7—C10—C11	108.8 (2)	H36B—C36—H36C	109.5
N7—C10—H10A	109.9	N9—C37—H37A	109.5
C11—C10—H10A	109.9	N9—C37—H37B	109.5
N7—C10—H10B	109.9	H37A—C37—H37B	109.5
C11—C10—H10B	109.9	N9—C37—H37C	109.5
H10A—C10—H10B	108.3	H37A—C37—H37C	109.5
N3—C11—N4	114.0 (3)	H37B—C37—H37C	109.5
N3—C11—C10	122.5 (3)		
			
N5—Mn1—O1—C28	−164.5 (2)	C5—C6—C7—C8	−1.7 (6)
N3—Mn1—O1—C28	−20.3 (3)	C6—C7—C8—C9	0.4 (5)
O2—Mn1—O1—C28	−0.4 (2)	C7—C8—C9—C4	2.1 (5)
N1—Mn1—O1—C28	90.5 (2)	C7—C8—C9—N1	−178.5 (3)
O3—Mn1—O1—C28	−83.8 (2)	N2—C4—C9—C8	178.4 (3)
N7—Mn1—O1—C28	140.3 (2)	C5—C4—C9—C8	−3.6 (5)
N5—Mn1—O2—C28	29.2 (3)	N2—C4—C9—N1	−1.1 (3)
O1—Mn1—O2—C28	0.3 (2)	C5—C4—C9—N1	176.9 (3)
N3—Mn1—O2—C28	169.2 (2)	C2—N1—C9—C8	−178.1 (3)
N1—Mn1—O2—C28	−89.6 (2)	Mn1—N1—C9—C8	27.5 (5)
O3—Mn1—O2—C28	90.9 (2)	C2—N1—C9—C4	1.2 (3)
N7—Mn1—O2—C28	−141.4 (2)	Mn1—N1—C9—C4	−153.1 (2)
N5—Mn1—O3—C32	−179.4 (4)	C1—N7—C10—C11	−76.5 (3)
O1—Mn1—O3—C32	87.7 (4)	C19—N7—C10—C11	156.7 (3)
N3—Mn1—O3—C32	−61.2 (4)	Mn1—N7—C10—C11	40.1 (3)
O2—Mn1—O3—C32	30.8 (4)	C18—N3—C11—N4	−1.1 (4)
N1—Mn1—O3—C32	26.9 (10)	Mn1—N3—C11—N4	154.6 (2)
N7—Mn1—O3—C32	−118.4 (4)	C18—N3—C11—C10	177.7 (3)
N5—Mn1—N1—C2	87.7 (2)	Mn1—N3—C11—C10	−26.6 (4)
O1—Mn1—N1—C2	−179.9 (2)	C13—N4—C11—N3	0.9 (4)
N3—Mn1—N1—C2	−32.5 (2)	C12—N4—C11—N3	179.8 (4)
O2—Mn1—N1—C2	−123.1 (2)	C13—N4—C11—C10	−177.9 (3)
O3—Mn1—N1—C2	−119.3 (8)	C12—N4—C11—C10	1.0 (6)
N7—Mn1—N1—C2	27.7 (2)	N7—C10—C11—N3	−13.2 (5)
N5—Mn1—N1—C9	−120.1 (3)	N7—C10—C11—N4	165.5 (3)
O1—Mn1—N1—C9	−27.6 (3)	C11—N4—C13—C14	178.6 (4)
N3—Mn1—N1—C9	119.7 (3)	C12—N4—C13—C14	−0.3 (7)
O2—Mn1—N1—C9	29.1 (3)	C11—N4—C13—C18	−0.3 (4)
O3—Mn1—N1—C9	32.9 (10)	C12—N4—C13—C18	−179.2 (4)
N7—Mn1—N1—C9	179.9 (3)	C18—C13—C14—C15	1.1 (6)
N5—Mn1—N3—C11	−16.8 (3)	N4—C13—C14—C15	−177.6 (4)
O1—Mn1—N3—C11	−156.5 (2)	C13—C14—C15—C16	−0.8 (8)
O2—Mn1—N3—C11	−173.1 (2)	C14—C15—C16—C17	0.5 (9)
N1—Mn1—N3—C11	95.9 (2)	C15—C16—C17—C18	−0.4 (8)
O3—Mn1—N3—C11	−90.9 (2)	C14—C13—C18—C17	−1.0 (6)
N7—Mn1—N3—C11	35.1 (2)	N4—C13—C18—C17	178.0 (4)
N5—Mn1—N3—C18	128.7 (3)	C14—C13—C18—N3	−179.3 (4)
O1—Mn1—N3—C18	−11.0 (4)	N4—C13—C18—N3	−0.3 (4)
O2—Mn1—N3—C18	−27.6 (3)	C16—C17—C18—C13	0.6 (6)
N1—Mn1—N3—C18	−118.6 (3)	C16—C17—C18—N3	178.5 (4)
O3—Mn1—N3—C18	54.7 (3)	C11—N3—C18—C13	0.8 (4)
N7—Mn1—N3—C18	−179.3 (3)	Mn1—N3—C18—C13	−146.8 (3)
O1—Mn1—N5—C20	−130.9 (2)	C11—N3—C18—C17	−177.2 (4)
N3—Mn1—N5—C20	70.5 (3)	Mn1—N3—C18—C17	35.2 (6)
O2—Mn1—N5—C20	−154.8 (2)	C10—N7—C19—C20	−80.3 (3)
N1—Mn1—N5—C20	−39.9 (2)	C1—N7—C19—C20	152.5 (3)
O3—Mn1—N5—C20	143.2 (2)	Mn1—N7—C19—C20	34.4 (3)
N7—Mn1—N5—C20	19.5 (2)	C27—N5—C20—N6	0.4 (4)
O1—Mn1—N5—C27	38.2 (3)	Mn1—N5—C20—N6	172.5 (2)
N3—Mn1—N5—C27	−120.3 (3)	C27—N5—C20—C19	−178.6 (3)
O2—Mn1—N5—C27	14.3 (4)	Mn1—N5—C20—C19	−6.6 (4)
N1—Mn1—N5—C27	129.3 (3)	C22—N6—C20—N5	−0.5 (4)
O3—Mn1—N5—C27	−47.7 (3)	C21—N6—C20—N5	179.3 (3)
N7—Mn1—N5—C27	−171.4 (3)	C22—N6—C20—C19	178.5 (3)
N5—Mn1—N7—C10	90.4 (2)	C21—N6—C20—C19	−1.7 (5)
O1—Mn1—N7—C10	151.6 (2)	N7—C19—C20—N5	−22.2 (4)
N3—Mn1—N7—C10	−40.06 (19)	N7—C19—C20—N6	158.9 (3)
O2—Mn1—N7—C10	−95.3 (2)	C20—N6—C22—C27	0.3 (4)
N1—Mn1—N7—C10	−153.0 (2)	C21—N6—C22—C27	−179.4 (3)
O3—Mn1—N7—C10	22.9 (2)	C20—N6—C22—C23	−179.8 (4)
N5—Mn1—N7—C1	−149.8 (2)	C21—N6—C22—C23	0.4 (6)
O1—Mn1—N7—C1	−88.5 (2)	N6—C22—C23—C24	−178.8 (4)
N3—Mn1—N7—C1	79.8 (2)	C27—C22—C23—C24	1.0 (6)
O2—Mn1—N7—C1	24.6 (3)	C22—C23—C24—C25	−0.8 (6)
N1—Mn1—N7—C1	−33.21 (19)	C23—C24—C25—C26	0.5 (7)
O3—Mn1—N7—C1	142.76 (19)	C24—C25—C26—C27	−0.3 (6)
N5—Mn1—N7—C19	−29.3 (2)	N6—C22—C27—C26	179.0 (3)
O1—Mn1—N7—C19	32.0 (3)	C23—C22—C27—C26	−0.9 (5)
N3—Mn1—N7—C19	−159.7 (2)	N6—C22—C27—N5	−0.1 (4)
O2—Mn1—N7—C19	145.1 (2)	C23—C22—C27—N5	−180.0 (3)
N1—Mn1—N7—C19	87.3 (2)	C25—C26—C27—C22	0.5 (5)
O3—Mn1—N7—C19	−96.7 (2)	C25—C26—C27—N5	179.4 (3)
C10—N7—C1—C2	149.6 (3)	C20—N5—C27—C22	−0.2 (4)
C19—N7—C1—C2	−83.3 (3)	Mn1—N5—C27—C22	−170.4 (2)
Mn1—N7—C1—C2	34.8 (3)	C20—N5—C27—C26	−179.1 (4)
C9—N1—C2—N2	−0.9 (3)	Mn1—N5—C27—C26	10.7 (5)
Mn1—N1—C2—N2	159.4 (2)	Mn1—O1—C28—O2	0.6 (4)
C9—N1—C2—C1	−179.6 (3)	Mn1—O1—C28—C29	−179.5 (4)
Mn1—N1—C2—C1	−19.3 (4)	Mn1—O2—C28—O1	−0.6 (4)
C4—N2—C2—N1	0.3 (3)	Mn1—O2—C28—C29	179.5 (4)
C3—N2—C2—N1	179.1 (3)	O1—C28—C29—C30	−168.8 (5)
C4—N2—C2—C1	179.0 (3)	O2—C28—C29—C30	11.1 (8)
C3—N2—C2—C1	−2.2 (5)	O1—C28—C29—C31	12.1 (7)
N7—C1—C2—N1	−13.6 (4)	O2—C28—C29—C31	−168.0 (5)
N7—C1—C2—N2	167.9 (3)	Mn1—O3—C32—N8	165.8 (4)
C2—N2—C4—C5	−177.2 (3)	C34'—N8—C32—O3	−32.7 (14)
C3—N2—C4—C5	3.9 (5)	C33—N8—C32—O3	−167.5 (7)
C2—N2—C4—C9	0.5 (3)	C33'—N8—C32—O3	173.4 (10)
C3—N2—C4—C9	−178.4 (3)	C34—N8—C32—O3	12.1 (9)
N2—C4—C5—C6	179.8 (3)	C36—N9—C35—O8	178.6 (8)
C9—C4—C5—C6	2.4 (5)	C37—N9—C35—O8	0.0 (11)
C4—C5—C6—C7	0.3 (5)		
